# The Prevalence of Soil-Transmitted Helminths and Associated Risk Factors among School Children at Sekela Primary School, Western Ethiopia

**DOI:** 10.1155/2020/8885734

**Published:** 2020-10-30

**Authors:** Addisu Tolera, Mebrate Dufera

**Affiliations:** ^1^Sekela Preparatory School, Horo Guduru Wollega Zone, Sekela, Ethiopia; ^2^Department of Biology, College of Natural and Computational Sciences, Wollega University, 395 Nekemte, Ethiopia

## Abstract

Soil-transmitted helminth (STH) infections are a major public health problem in tropical and developing countries in relation to poverty, inadequate hygiene, and sanitation. This study was aimed at assessing the status of STH and associated risk factors among school children in the case of Sekela primary school. Cross-sectional descriptive studies were conducted in May 2019 and from 384 children, both males and females of equal proportion were used. A stool sample was collected randomly and examined in the laboratory under a microscope. The quantitative data were analyzed using SPSS version 20. The difference was considered statistically significant at *p* value = 0.05. The overall STH infections observed in the study area were about 25.78% and were moderate transmission. The predominant helminth was *A. lumbricoides* (9.86%) followed by hookworm 6.25%, *T. trichiura* 5%, *H. nana* 3.10%, and *H. diminuta* 1.56%. Infections were generally more in males than females, in which 15.36% males and 10.41% females. In the case of age group, age groups between 7-8 years were more infected (13.28%). Infection rate decreases with increasing ages. Multivariate logistic regression analysis result indicated that wearing shoes, hand washing practice, family member, and residence were found to be associated risk factors for STH infections. Being urban dwellers and having family members with less than 2 children were found to be preventive. Lack of latrine, playing barefoot, untrimmed fingernail, eating raw vegetables, and absence of hand washing were major risk factors. In conclusion, the study showed that there was moderate transmission of infection among the study participants. Community-based health education using media, morbidity control through deworming, and improving sanitation should be strengthened as a measurement to control the transmission rate.

## 1. Introduction

Soil-transmitted helminth (STH) infections are among the most widespread of all chronic human infections. Low standards of living, poor socioeconomic status, poor personal hygiene, and poor environmental sanitation are considered as the major causes of STH infections [1]. In 2010, the World Health Organization (WHO) reported these infectious diseases as part of the portfolio of major neglected tropical diseases worldwide [2]. Maximum worm burdens occur in human populations at 5-10 years of age [3]. The effects of worm infection including nutritional deficiencies which aggravate malnutrition and worsen the rates of anemia and impaired physical and mental development contributed significantly to school absenteeism [4].

The most common STHs which are found worldwide are *Trichuris trichiura*, *Ascaris lumbricoides*, and the hookworms (*Necator americanus* and *Ancylostoma duodenale*), and they stand out because of their widespread prevalence and distribution that result in hundreds of millions of human infections [5], with the greatest public health burden occurring in developing countries, particularly in sub-Saharan Africa [6]. Infection with *T. trichiura* (trichuriasis) is the third most common helminth infections of humans [7]. The distribution of trichuriasis is worldwide, being most abundant in the warm moist regions of the world. It affects about 1 billion people throughout the world. In Ethiopia, there are about 21 million people infected with this parasite, which accounts 13% of the disease burden in sub-Saharan Africa in which high prevalence occurs in areas with tropical weather and poor sanitation practices [8]. *T. trichiura* was found in more than 90% of 50 communities, with a mean prevalence of 49% [9]. In Ethiopia, prevalence of trichuriasis among school-age children reported at 86.4% in Wondo Genet [10], 9.5% in South Gondar [11], and 47.6% in Jimma town [12].

Globally, ascariasis prevalence is estimated to be more than 1.3 billion, approximately one-quarter of the world population [13], and over 250 million suffer from associated morbidity. It is estimated that 204 million cases of ascariasis occur in East Asia and the Pacific, 140 million in India, 97 million in South Asia, 84 million in Latin America and the Caribbean, 23 million in the Middle East and North Africa, and 173 million in sub-Saharan Africa including Ethiopia [14].

The soil-transmitted helminths, including *Ascaris lumbricoides*, *Trichuris trichiura*, *Ancylostoma duodenale*, and *Necator americanus*, occur in single or mixed infections, with at least two species of parasites present in the same individual. These are found in more than one billion people in sub-Saharan Africa, Latin America, China, and East Asia [15].

Hookworms are amongst the most widespread of soil-transmitted intestinal helminth parasites [16]. Infection in humans is caused by an infection with *Necator americanus* and *Ancylostoma duodenale* and is transmitted through contact with contaminated soil.

An estimated 1.2 billion people worldwide have been infected [17], mainly in the tropics and subtropics, with the foci predominantly in areas of rural poverty within Asia, sub-Saharan Africa, and Latin America [14]. In Ethiopia, it is estimated that there are 11 million people infected with hookworm, which makes the country to have the third-highest burden rank in sub-Saharan Africa [8].

The majority of detection methods routinely used for the diagnosis of intestinal helminths and protozoan infections in humans are inexpensive and simple to perform [18]. However, these tests have limitations, particularly regarding their sensitivity. Thus, the use of more than one method is necessary to detect different parasitic forms, especially under conditions of low parasite burdens [19]. Since the early 20th century, the detection of intestinal parasites has improved with the development of several techniques for parasitic structures recovery and identification, which differ in sensitivity, specificity, practicality, cost, and infrastructure demand [20]. In Europe, fresh, nonpreserved stool specimens are generally used for examination. Because intestinal parasites are shed intermittently, patients are asked to deliver multiple stool samples for examination [21]. The TFT had the highest positivity rates for *Ascaris lumbricoides* (58.1%) and hookworm (75%); the HPJ technique had the highest positivity rate for *Strongyloides stercoralis* (50%). Although a combination of tests is the most accurate method for the diagnosis of internal parasites, the TFT reliably estimates the prevalence of protozoa and selected helminths, such as *A. lumbricoides* and hookworm. Further studies are needed to evaluate the detection accuracy of the TFT in samples with varying numbers of parasites [22].

World Health Organization recommends a baseline survey in schoolchildren to determine the prevalence and intensity of infections before the implementation of a treatment program. In Ethiopia, although several studies had revealed that intestinal parasite infections are widely distributed with high prevalence rates, there was no previous research conducted at Sekela primary school concerning STH infections. Therefore, the main focus of this study was to assess the status of these parasites and associated risk factors among school children and to initiate control and prevention methods in the study area.

## 2. Materials and Methods

### 2.1. Study Area

This study was conducted in Sekela town, at Sekela primary school. The town is located in Horro Guduru Wollega, Western Ethiopia. It is located 331 Km northern west of Addis Ababa. The Sekela town is located at 9°34′N latitude and 37°06′E longitude and at an altitude of 2200-3100 mas. The mean annual temperature of the town was 20°C.The annual rainfall range from 1500-2000 mm. The climate of the District and its surroundings are categorized as Dega (cool zone). The soil is mainly loam. Farming in the study area is a mixed crop-livestock production system and is an integral component of the traditional farming system of Ethiopia. The major crops grown in the area are sorghum, wheat, teff, barley, maize, and others. According to the Sekela town administration office, the total population number in 2018 was estimated that 23,330.

### 2.2. Study Population

The study population was primary school children at Sekela Town. Among the total population of 632 (male 334 and female 298) subjects, 384 (male 192 and female 192) with the age range of 7 to 15 years were included in the study.

### 2.3. Study Design

A cross-sectional descriptive study was conducted to identify the prevalence of soil-transmitted helminths and associated risk factors among Sekela primary school.

### 2.4. Sample Size Determination and Sampling Techniques

Calculation of sample size (*n*) was done using the formula for estimating single proportion (*n* = *Z*^2^ *P* (1 − *P*)/*d*2), where *n* is sample size, *d* is worst accepted value/marginal error, *Z* is statistic value for level of 95% confidence, is 1.96; *P* is expected prevalence or proportion which is 0.5. However, since there were no previous studies conducted in the area, 50% was assumed for prevalence (*P*). A minimum of 384 samples (*n*) was generated using a 5% marginal error (*d*). To determine the proportion of children who participated in the study, schoolchildren were selected with a simple random sampling technique. Finally, children from each grade and section were selected with systematic random sampling proportionally by dividing the total sample size.

### 2.5. Research Methodology

The study utilized descriptive research methods. The children selected by lottery method of simple random sampling technique, out of the total number of school children, male 192 and female 192, the total selected sample size was *n* = 384. The selected children were sent to the health center and gave their stool sample to a laboratory technician and examined, then positive stool was identified and recorded.

A fresh stool of 0.5 g sample was collected from each child selected from the school using labeled clean, dry, and wide-mouthed stool cups. The samples were transported to the Sekela health center for parasitological examination and were identified as positive or negative then recorded according to a given code number.

### 2.6. Data Collection

In this study, data gathering instruments both structured questionnaires and parasitological examination were used.

#### 2.6.1. Questionnaire Survey

The questionnaire was pretested in communities with similar characteristics for the necessary adjustment to be made. Structured questionnaires were prepared in English and translated to Afan Oromo to distribute to trained data collectors. Sociodemographic characteristics and risk factors that predispose to STH infection were collected using the questionnaire.

#### 2.6.2. Stool Sample Collection and Parasitological Examination

After completing the questionnaire survey, the individuals who agreed to participate in the study were provided with marked clean labeled plastic stool cups and instructed to bring approximately 5 grams of their own stool. The samples were examined for the detection and identification of intestinal parasite at facility setting by two laboratory technologists using double direct saline techniques within 30 minutes of collection, and stool samples were considered positive if the intestinal parasites were detected.

### 2.7. Statistical Analysis

Data were entered on a computer, and validation was performed in Microsoft Excel 2007 spreadsheets and transferred into SPSS version 20.0 software for statistical analysis. The prevalence of STH calculated as the percentage of number of children was found positive for any STH species to the total number of children who provided complete data. Multivariate logistic regression was used to identify the associations of STH infections with independent variables and considered statistically significant with *p* value < 0.05.

## 3. Results

### 3.1. Sociodemographic Factors

The prevalence of soil-transmitted helminth infections survey was conducted at Sekela primary school. The school is located in Sekela town. But the children came to the school from the town and neighboring rural villages with a total sample size of 384. All samples were analyzed to estimate the prevalence of soil-transmitted helminths and associated risk factors in the study area. The equal number of study participants, male and female, from urban and rural areas were used (Table 1).

### 3.2. Prevalence of STH Infections

Out of 384 stool specimens collected and examined, 99 (25.78%) were found positive for one helminth infection. Five species: *A. lumbricoides*, hookworm, *T. trichiura*, *H. nana*, and *H. diminuta* of soil-transmitted helminth infections were identified in the study. *A. lumbricoides* was the first predominate parasite detected in 38 (9.89%) followed by hookworm 24 (6.25%) and *T. trichiura* 19 (5%). Additionally, *H. nana* and *H. diminuta* were also detected in 12 (3.10%) and 6 (1.56%), respectively. Males 60 (15.62%) were mostly affected than females 39 (10.15%) (Table 2).

**Figure 1:**
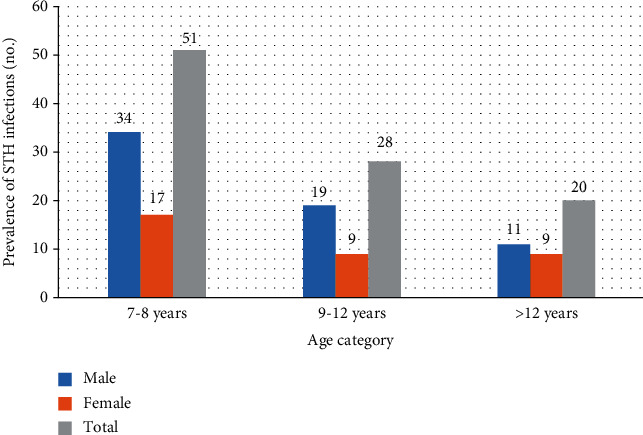
Prevalence of STH infections with their age at Sekela primary school in 2019.

The prevalence of STH infections was highest 51 (13.28%) in age case 7-8 years followed by 28 (7.29%) in 9-12 years and least 20 (5.2%) in age group above 12 years (Figure 1).

Children came from rural villages, 41 (10.67%) males and 27 (7.03%) females were positive in one of soil-transmitted helminth infections, while children from urban Sekela town, males 19 (6.77%) and 12 (3%) females, were positive in one of soil-transmitted helminth infections. In both rural and urban cases, males were more infected than females (Table 3).

Multivariate logistic regression result risk factors show that nonlatrine use was found to be associated risk factors for STH infections. Accordingly, the odds of STH infections in this primary school children were about six times higher in those who do not use latrine than those who use latrine (AOR = 5.95% CI 1.66-13.6, *p* value = 0.006). The odds of STH infections in the study subject were four times higher in those who do not wash their hands than those who wash their hands (AOR = 3.96%CI = 0.77 − 20.44, *p* ≤ 0.001). Residential place (urban) and trimming fingernail were the prevention of STH infections in this study. Regarding the residence of the study participants, living in urban was the preventive factor for the STH infections. Living in urban prevents STH by 0.93% when compared to their counterparts (AOR = 0.068, 95% CI, 0.018-0.25, *p* ≤ 0.001). Trimming fingernail prevents STH infections by 0.67% when compared to those untrimming fingernail (AOR = 0.322, 95% CI = 0.152 − 0.682, *p* = 0.003). Family having more lifestyle prevent soil-transmitted helminth infections by 0.83% than those having less living family style (noneducated) (AOR = 0.166, 95% CI = 0.039 − 0.711, *p* = 0.016) (Table 4).

## 4. Discussions

STH infections are infections of the intestine, which is transmitted by contact with contaminated eggs and larvae of the worm that enter the body through contaminated food and by penetrating the skin. The infection was worldwide mostly tropical and subtropical area especially affects preschool children and school children. In the present study, the overall prevalence of STH infections found in Sekela primary school was 99 (25.73%). Based on MDA applications, WHO STH endemic areas are classified into three categories (high transmission, where prevalence is >50%; moderate transmission, where prevalence is between 20%-50%; and low transmission, where prevalence is <20%) [23]. Accordingly, the study area would be classified into the moderate transmission group calling for annual deworming. About 59 (15.36%) males and 40 (10.41%) females were infected by one of the helminths. Males shared more infection as compared to females which is contrary to studies conducted by [24]. The difference may be attributed to the study design (facility-based versus community-based).

Regarding age group, age group between 7-8 years was more infected (13.28%) followed by 9-12 years (7.29%) and above 12 years (5.2%) which is in line with studies conducted by [25], in which the prevalence of intestinal helminth infection was significantly higher in children of ages 10 to 14 years than children of ages 5 to 9 years. The prevalence of STH infections in the present study decreases with the age and also in agreement with studies conducted by [26].

Concerning residential issues, 31 (8.07%) infected cases came from Sekela town (urban), while 68 (17.70%) were from the surrounding villages (rural). The difference was 9% suggesting children in rural area have more chances to be contaminated due to poor sanitation, lack of latrine, and poor hand washing practice and different from studies conducted by [25], in which *A. lumbricoides* and *T. trichiura* infections were slightly more common among children in urban areas.

The prevalence of *A. lumbricoides* infection in the present study (9.86%) was much less than previous studies conducted in various parts of Ethiopia; 39.8% in North Gondar [27], 83.4% in Wendo Genet [10], 39.5% in Jimma Town [12], and 37.2% in Bushilo village [28]. The prevalence of hookworm in the present study was 6.25% contrary to studies conducted in different parts of Ethiopia; 6.7% in Eastern [29] and 22% in North West [30] and the national prevalence 16% [8]. *T. trichiura* is another STH infection with 5% and was much lower than previous studies conducted in Ethiopia; 9.5% in south Gondar [11], 86.4% in Wondo Genet [10], 47.6% in Jimma town [12], and 49% national prevalence of *T. trichiura* [9]. In general in Ethiopia, possible reasons for the reduction of the prevalence of STH infections could be improved sanitation, health education, and morbidity control through deworming. The limitation of the present study was the lack of Triple Faces Test (TFT) contrary to a study conducted by [21], in which TFT is an effective method for the detection of intestinal parasites in stool samples in routine clinical practice and also it is of fundamental importance for the evaluation of sanitation interventions for the control of internal parasitosis as reported by [22].

### 4.1. Conclusion and Recommendation

STH infections are among the most prevalent and affect the poorest and most deprived communities. In Ethiopia, the disease affects millions of people. The present study showed that the prevalence of soil-transmitted helminth infection was with moderate transmission. The three major soil-transmitted helminths were *A. lumbricoides*, hookworm, and *T. trichiura*. Results indicated that wearing shoes, hand washing practice, family member, and residence were found to be the major-associated risk factors, while being urban dwellers and having family member less than 2 children were found to be preventive. In routine clinical practice, since Triple Faces Test is an effective method for the detection of intestinal parasites in stool samples, the technical procedures used in fecal processing for diagnosis of intestinal parasites should be further investigated. Community-based health education using media, morbidity control through deworming, and improving water and food sanitation should be strengthened as a measurement to control the transmission rate.

## Figures and Tables

**Table 1:** Sociodemographic characteristic of the respondents at Sekela primary school (*n* = 384) in 2019.

Gender	Age			Educational level				Residence	
	7-8	9-12	>12	Grade 1	Grade 2	Grade 3	Grade 4	Urban	Rural
Male	80	80	32	40	40	80	32	96	96
Female	80	80	32	40	40	80	32	96	96

**Table 2:** STH infections in children at Sekela primary school by sex (*n* = 384) in 2019.

Study subjects (*n* = 384)	*A. lumbricoidesn* (%)	Hookworm *n* (%)	*T. trichiuran* (%)	*H. nanan* (%)	*H. diminutan* (%)	Total *n* (%)
Male	24 (6.25%)	14 (3.64%)	12 (3.12%)	6 (1.56%)0	4 (1%)	60 (15.62%)
Female	14 (3.64%)	10 (2.6%)	7 (1.82%)	6 (1.56%)	2 (0.56%)	39 (10.15%)
Total	38 (9.89%)	24 (6.25%)	19 (5%)	12 (3.12%)	6 (1.56%)	99 (25.78%)

**Table 3:** Prevalence of STH infections by residence (rural and urban) at Sekela primary school in 2019.

Parasite species		Study subjects (*n* = 384)							
		Male (*n* = 192)				Female (*n* = 192)			
	Age	7-8 *n* = 40	9-12 *n* = 40	>12 *n* = 16	Total 96	7-8 *n* = 40	9-12 *n* = 40	>12 *n* = 16	Total *n* = 96
*A. lumbricoides*	Rural	7 (1.8%)	5 (1.3%)	3 (0.78%)	15 (3.9%)	4 (1%)	3 (0.78%)	4 (1%)	11 (2.86%)
	Urban	3 (0.78%)	4 (1%)	2 (0.52%)	9 (2.34%)	2 (0.5%)	1 (0.26%)	0%	3 (0.78%)
Hookworm	Rural	5 (1.3%)	2 (0.56%)	3 (0.78%)	10 (2.86%)	4 (1%)	1 (0.56%)	2 (0.78%)	7 (1.82%)
	Urban	2 (0.52%)	2 (0.52%)	0%	4 (1%)	1 (1%)	1 (1%)	1 (1%)	3 (0.7%)
*T. trichiura*	Rural	4 (1%)	2 (0.56%)	4 (1%)	10 (2.6%)	2 (0.52%)	1 (0.26%)	1 (0.26%)	4 (1.04%)
	Urban	1 (0.5%)	0%	1 (0.26%)	2 (0.5%)	1 (0.2%)	1 (0.26%)	1 (0.26%)	3 (0.78%)
*H. nana*	Rural	1 (0.26%)	1 (0.26%)	2% (0.52)	4 (1%)	2 (0.26%)	1 (0.26%)	0%	3 (0.78%)
	Urban	1 (0.26%)	2 (0.52%)	(0%)	3 (0.78%)	1 (0.2%)	2 (0.56%)	(0%)	3 (0.78%)
*H. diminuta*	Rural	1 (0.26%)	1 (0.26%)	0%	2 (0.56%)	2 (0.26%)	0%	0%	2 (0.56%)
	Urban	1 (0.26%)	0%	0%	0%	0%	0%	0%	0%
Total	Rural	18 (4.16%)	11 (2.86%)	12 (3.12%)	41 (10.67%)	14 (3.64%)	6 (1.56%)	7 (1.8%)	27 (7.03%)
	Urban	8 (2%)	8 (2%)	3 (0.78%)	19 (6.77%)	5 (1. %)	5 (1.3%)	2 (0.56%)	12 (3%)

**Table 4:** Major risk factors and their associations in multivariate regression analysis at Sekela primary school in 2019.

Risk factors	Exp (B)	95% CI		*p*
		Lower boundary	Upper boundary	
Nonlatrine use	5.95	1.62	13.6	0.004
Nonhand washing practice	3.96	0.771	20.4	0.000
Trimming fingernail	0.322	0.152	0.682	0.003
Wearing shoes habit	5.58	2.35	13.27	0.000
Eating nonraw vegetable	0.263	0.096	0.723	0.010
Family background	0.166	0.039	0. 711	0.016
Children not living with parents	3	1.19	7.71	0.020
Living at urban	0.068	0.018	0.25	0.001
Family member <2 than 2	0.77	0.006	0.994	0.04

## Data Availability

The data set generated from patients' clinical record is not publicly available to protect patient confidentiality. Unidentifiable data can be obtained from the corresponding author upon reasonable request.

## References

[1] Bundy D. A. P., Guyatt H. L. (1996). Schools for health: focus on health, education and the school-age child. *Parasitology Today*.

[2] WHO (2010). *First WHO report on neglected tropical diseases*.

[3] Bundy D. A. P., Chan M. S., Savioli L. (1995). Hookworm infection in pregnancy. *Transactions of the Royal Society of Tropical Medicine and Hygiene*.

[4] Saathoff E., Olsen A., Kvalsvig J. D., Appleton C. C. (2004). Patterns of geohelminth infection, impact of albendazole treatment and re-infection after treatment in schoolchildren from rural KwaZulu-Natal/South-Africa. *BMC Infectious Diseases*.

[5] Pullan R. L., Brooker S. J. (2012). The global limits and population at risk of soil-transmitted helminth infections in 2010. *Parasites & Vectors*.

[6] Bethony J., Brooker S., Albonico M. (2006). Soil-transmitted helminth infections: ascariasis, trichuriasis and hookworm. *Lancet*.

[7] Peters W., Pasvol G. (2005). *Atlas of Tropical Medicine and Parasitology*.

[8] Tadesse Z., Hailemariam A., Kolaczinski J. H. (2008). Potential for integrated control of neglected tropical diseases in Ethiopia. *Transactions of the Royal Society of Tropical Medicine and Hygiene*.

[9] Belete H., Kloos H., Yemane B., Damen H., Kloos H. (2006). *Intestinal parasitism, the epidemiology and ecology of health and disease in Ethiopia*.

[10] Erko B., Medhin G. (2003). Human helminthiasis in Wondo Genet, southern Ethiopia, with emphasis on geohelminthiasis. *Ethiopian medical journal*.

[11] Jemaneh L. (2000). The epidemiology of Schistsoma mansoni and soil-transmitted helminths in elementary school children from the South Gondar Zone of Amhara National Regional State. *Ethiopia Ethiopian Mededical Journal*.

[12] Debalke S., Worku A., Jahur N., Mekonnen Z. (2013). Soil Transmitted Helminths and Associated Factors among Schoolchildren in Government and Private Primary School in Jimma Town, Southwest Ethiopia. *Ethiopian Journal of Health Sciences*.

[13] Crompton D. W. T. (1999). How much human helminthiasis is there in the world?. *The Journal of Parasitology*.

[14] de Silva N. R. (2003). Impact of mass chemotherapy on the morbidity due to soil-transmitted nematodes. *Acta Tropica*.

[15] Chammartin F., Scholte R. G. C., Guimarães L. H., Tanner M., Utzinger J., Vounatsou P. (2013). Soil-transmitted helminth infection in South America: a systematic review and geostatistical meta-analysis. *The Lancet Infectious Diseases*.

[16] Chan L., Bundy D. A. P., Kan S. P. (1994). Aggregation and predisposition to Ascaris lumbricoides and Trichuris trichiura at the familial level. *Transactions of the Royal Society of Tropical Medicine and Hygiene*.

[17] Chan M. S. (1997). The global burden of intestinal nematode infections: fifty years on. *Parasitology Today*.

[18] De Carli G. A. (2001). *Parasitologia clínica: seleção de métodos e técnicas de laboratório para diagnóstico das parasitoses humanas*.

[19] Peralta R. H. S., Peralta J. M. (2007). Diagnóstico da estrongiloidíase humana: importância e técnicas. *Revista de Patologia Tropical*.

[20] Soares F. A., Benitez A. d. N., Santos B. M. d. (2020). A historical review of the techniques of recovery of parasites for their detection in human stools. *Revista da Sociedade Brasileira de Medicina Tropical*.

[21] van Gool T., Weijts R., Lommerse E., Mank T. G. (2003). Triple Faeces Test: an effective tool for detection of intestinal parasites in routine clinical practice. *European Journal of Clinical Microbiology & Infectious Diseases*.

[22] de Carvalho G. L. X., Moreira L. E., Pena J. L., Marinho C. C., Bahia M. T., Machado-Coelho G. L. L. (2012). A comparative study of the TF-Test®, Kato-Katz, Hoffman-Pons-Janer, Willis and Baermann-Moraes coprologic methods for the detection of human parasitosis. *Memórias do Instituto Oswaldo Cruz*.

[23] World Health Organization (2002). *Prevention and control of schistosomiasis and Soil transmitted helminthiasis, WHO Technical Report Series, 912*.

[24] Tekalign E., Bajiro M., Ayana M., Tiruneh A., Belay T. (2019). Prevalence and Intensity of Soil-Transmitted Helminth Infection among Rural Community of Southwest Ethiopia: A Community-Based Study. *BioMed Research International*.

[25] Alelign T., Degarege A., Erko B. (2015). Soil-Transmitted Helminth Infections and Associated Risk Factors among Schoolchildren in Durbete Town, Northwestern Ethiopia. *Journal of Parasitology Research*.

[26] Stothard J. R., Imison E., French M. D., Sousa-figueiredo J. C., Khamis I. S., Rollinson D. (2008). Soil-transmitted helminthiasis among mothers and their pre-school children on Unguja Island, Zanzibar with emphasis upon ascariasis. *Parasitology*.

[27] Mathewos B., Alemu A., Woldeyohannes D. (2014). Current status of soil transmitted helminths and Schistosoma mansoni infection among children in two primary schools in North Gondar, Northwest Ethiopia: a cross sectional study. *BMC Research Notes*.

[28] Terefe A., Shimelis T., Mengistu M., Hailu A., Erko B. (2011). Schistosomiasis mansoni and soil-transmitted helminthiasis in Bushulo village, southern Ethiopia. *Ethiopian Journal of Health Development*.

[29] Tadesse G. (2005). The prevalence of intestinal helminthic infections and associated risk factors among school children in Babile town, eastern Ethiopia. *Ethiopian Journal of Health Development*.

[30] Hailu T., Yimer M. (2014). Prevalence of Schistosoma mansoni and geo-helminthic infections among patients examined at Workemeda Health Center, Northwest Ethiopia. *Journal of Parasitology and Vector Biology*.

